# P-321. Risk Factors for Methicillin-Resistant *Staphylococcus aureus* Carriers in the Intensive Care Unit: A Single-Center, Retrospective Cohort Study in Japan

**DOI:** 10.1093/ofid/ofae631.524

**Published:** 2025-01-29

**Authors:** Hisato Yoshida, Masayuki Nigo, Hiromichi Iwasaki

**Affiliations:** University of Fukui, Yoshida-gun, Fukui, Japan; Houston Methodist Hospital, Houston, Texas; University of Fukui Hospital, fukui, Fukui, Japan

## Abstract

**Background:**

Methicillin-resistant *Staphylococcus aureus* (MRSA) is one of the most common pathogens in the intensive care unit (ICU). Active surveillance cultures (ASCs) for MRSA often performed in ICUs may not be optimal in ICU settings with low prevalence of MRSA. This study aims to determine the risk factors of MRSA carriage in the ICU and develop a clinical predictive model to optimize the screening process.

**Methods:**

All patients screened for MRSA by nasal ASC at the time of ICU admission between April 2015 and August 2022 were retrospectively included and divided into MRSA-positive and MRSA-negative groups. Patients’ characteristics were evaluated to determine the prevalence of MRSA and the risk factors. Cost analysis was conducted based on the risk factors identified by our analysis.

**Results:**

Of the 3,927 ICU patients included, 133 (3.4%) were MRSA-positive. Multivariate analyses showed that risk factors for MRSA carriage were age ≥60 years (odds ratio [OR]: 1.60), history of hospitalization within a year (OR: 1.51), admission under departments dealing with skin and soft tissues (OR: 3.00), and ICD-10 codes classification I (OR: 3.44). Screening patients based on at least one of the risk factors exhibited high sensitivity (93.2%) to identifying MRSA carriage and could reduce ASC overall costs by 86.9%.

**Conclusion:**

This study suggests that universal ASCs to detect MRSA may not be optimal in ICU settings with low prevalence of MRSA. Targeted screening based on risk factors may reduce the volume and cost of MRSA screening. Prospective studies are warranted to confirm these findings.

**Disclosures:**

**All Authors**: No reported disclosures

